# Supplementary material for: Risk Factors Associated with Mechanical Ventilation in Critical Bronchiolitis
Source: Children (Basel). 2021 Nov 11;8(11):1035. doi: 10.3390/children8111035 (PMC8618830; doi:10.3390/children8111035)
Supplement: Supplementary file 1 [file children-08-01035-s001.zip › FINAL SUPPL.pdf]

Supplementary Tables

Table S1. Patient Demographics for Early Intubation Group only (n = 96).

	**Prior to admission (52)**	**After admission (44)**	$p^{\dagger}$
Age at PICU admission			
Median (IQR), mo	2.5 (1.5-8)	3 (1.5-4.75)	0.64
n (%)			
≤1 month	9 (17)	7 (16)	0.13
2-12 months	34 (65)	35 (69)	
13-24 months	9 (17)	2 (11)	
Weight (kg), median (IQR)	5.6 (3.4-7.9)	5.2 (4.5-6.6)	0.82
Sex, *n* (%)			
Female	27 (52)	19 (43)	0.39
Ethnicity, *n* (%)			
Hispanic	7 (13)	7 (16)	0.73
Non-Hispanic	45 (87)	37 (84)	
Race, *n* (%)			
Caucasian	31 (60)	21 (48)	0.24
Non-Caucasian	21 (40)	23 (52)	
Insurance, *n* (%)			
Medicaid	26 (50)	17 (39)	0.26
Other	26 (50)	27 (61)	

PICU = pediatric intensive care unit, IQR = interquartile range, mo = months; † Continuous variables are presented as median (interquartile range) and compared using the Wilcoxon rank-sum test; categorical variables are presented as *n* (%) and compared using the chi-square test of independence.

Table S2. Clinical Characteristics for Early Intubation Group only (n = 96).

	**Prior to admission (52)**	**After admission (44)**	$p^{\dagger}$
Gestational age at delivery			
Median (IQR), week	37 (34-38)	36 (33-38)	0.62
n (%)			
Full-term at birth	27 (52)	21 (48)	0.68
Pre-term at birth	25 (48)	23 (52)	
Late (33 to <37 weeks)	15 (29)	13 (29)	0.56
Very (28 to <32 weeks)	7 (13)	6 (14)	
Extreme (<28 weeks)	3 (6)	4 (9)	
Pre-exiting genetic conditions, *n* (%)	4 (8)	4 (9)	0.80
Pre-existing neurologic conditions, *n* (%)	10 (19)	7 (16)	0.67

IQR = interquartile range; † Continuous variables are presented as median (interquartile range) and compared using the Wilcoxon rank-sum test; categorical variables are presented as *n* (%) and compared using the chi-square test of independence.

Table S3. Hospital Course and Pathogen Data for Early Intubation Group only (n = 96).

	**Prior to admission (*n* = 52)**	**After admission (*n* = 44)**	$p^{\dagger}$
Intubation, *n* (%)	52 (54)	44 (46)	-
Duration (d), median (IQR)	5 (4-8)	7 (6-10)	**0.002**
Ventilator-free-days, median (IQR)	23 (20-24)	21 (18-22)	**0.002**
PICU LOS (d), median (IQR)	6 (4-9)	8 (6.25-10)	**0.005**
PIM-III ROM, % (IQR)	1.4 (1-2.5)	1.1 (0.9-1.3)	0.07
Vasoactives usage, *n* (%)	5 (10)	2 (5)	0.34
Mortality, *n* (%)	2 (4)	0 (0)	0.12
Pathogen^{f} (#), median (IQR)	2 (1-3)	2 (1.25-3)	0.35
Pathogen positive, *n* (%)	51 (98)	44 (100)	0.36
Single	14 (27)	11 (20)	0.79
Multiple	37 (73)	33 (75)	
Virus only	18 (35)	11 (25)	0.47
Bacterial only	1 (2)	2 (5)	
Virus + Bacteria	32 (63)	31 (70)	
Viral count (>1)	11 (22)	7 (16)	0.48

IQR = interquartile range, d = days, PICU LOS = pediatric intensive care unit length of stay, PIM-III ROM = Pediatric Index of Mortality-III Risk of Mortality, # = number; ^{f} Refer to Table S5 for details; † Continuous variables are presented as median (interquartile range) and compared using the Wilcoxon rank-sum test; categorical variables are presented as *n* (%) and compared using the chi-square test of independence. Bold highlights p values < 0.05.

Table S4. Infecting Pathogen Characteristics (all groups, *n* = 573).

	NRS (440)	IMV (133)	$p^{\dagger}$	Early IMV (96)	Late IMV (37)	$p^{\dagger}$
Viral, median (IQR), *n* (%)	1 (1-1)	1 (1-1)	0.29	1 (1-1)	1 (1-2)	0.24
Respiratory syncytial virus	261 (64)	88 (67)		60 (63)	28 (78)	
Rhino/enterovirus	146 (36)	42 (32)		29 (31)	13 (36)	
Human metapneumovirus	31 (8)	5 (4)		4 (4)	1 (3)	
Coronavirus	26 (6)	7 (5)		4 (4)	3 (8)	
Parainfluenza	19 (5)	12 (9)		9 (9)	3 (8)	
Adenovirus	16 (4)	7 (5)		5 (5)	2 (6)	
Influenza A	3 (1)	1 (1)		1 (1)	0 (0)	
Cytomegalovirus	0 (0)	1 (1)		1 (1)	0 (0)	
Bacterial, median (IQR), *n* (%)	0 (0-0)	1 (0-2)	-	1 (0-1.8)	1 (1-2)	**0.025**
Hemophilus influenzae	n.c.	54 (41)		39 (41)	15 (42)	
Moraxella catarrhalis	n.c.	35 (27)		21 (22)	14 (39)	
Streptococcus pneumoniae	n.c.	20 (15)		10 (11)	10 (28)	
Staphylococcus aureus	1 (0)	19 (15)		13 (14)	6 (17)	
Pseudomonas aeruginosa	n.c.	6 (5)		2 (2)	4 (11)	
Klebsiella sp.	n.c.	4 (3)		4 (4)	0 (0)	
Chryseobacterium indologenes	n.c.	2 (2)		2 (2)	0 (0)	
Escherichia coli	n.c.	2 (2)		0 (0)	2 (6)	
Acinetobacter sp.	n.c.	1 (1)		1 (1)	0 (0)	
Neisseria meningitidis	n.c.	1 (1)		0 (0)	1 (3)	
Group A streptococcus sp.	n.c.	1 (1)		0 (0)	1 (3)	
Mycoplasma pneumoniae	6 (1)	0 (0)		0 (0)	0 (0)	

IQR = interquartile range, n.c.= not checked; † Continuous variables are presented as median (interquartile range) and compared using the Wilcoxon rank-sum test; categorical variables are presented as *n* (%) and compared using the chi-square test of independence. Bold highlights *p* values < 0.05.

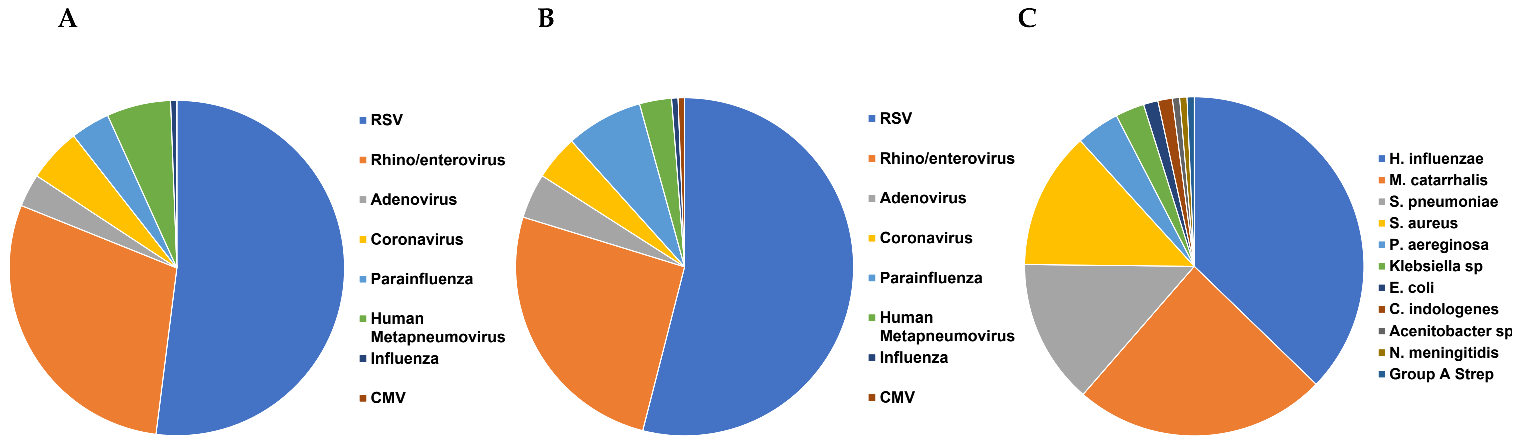

Figure S1. Infecting pathogens presented in Table S4. (A) Viral pathogens isolated from the NRS group. (B) Viral pathogens isolated from the IMV group. (C) Bacterial pathogens isolated from the IMV group.

Table S5. Infecting Pathogen Characteristics for Early Intubation Group only (*n* = 96).

	**Prior to admission (*n* = 52)**	**After admission (*n* = 44)**	***p***[†]
Viral, median (IQR), *n* (%)	1 (1-1)	1 (1-1)	0.51
Respiratory syncytial virus	29 (57)	31 (70)	
Rhino/enterovirus	17 (33)	12 (27)	
Human metapneumovirus	5 (10)	0 (0)	
Coronavirus	2 (4)	2 (5)	
Parainfluenza	6 (12)	3 (7)	
Adenovirus	3 (6)	1 (2)	
Influenza A	1 (2)	0 (0)	
Cytomegalovirus	0 (0)	1 (2)	
Bacterial, median (IQR), *n* (%)	1 (0-1)	1 (0.25-2)	0.12
Hemophilus influenzae	19 (37)	20 (45)	
Moraxella catarrhalis	6 (12)	7 (16)	
Streptococcus pneumoniae	11 (22)	10 (23)	
Staphylococcus aureus	4 (8)	6 (14)	
Pseudomonas aeruginosa	0 (0)	4 (9)	
Klebsiella sp.	0 (0)	2 (5)	
Chryseobacterium indologenes	2 (4)	0 (0)	
Escherichia coli	1 (2)	0 (0)	
Acinetobacter sp.	1 (2)	0 (0)	
Neisseria meningitidis	0 (0)	0 (0)	
Group A streptococcus sp.	0 (0)	0 (0)	
Mycoplasma pneumoniae	0 (0)	0 (0)	

IQR = interquartile range; [†] Continuous variables are presented as median (interquartile range) and compared using the Wilcoxon rank-sum test; categorical variables are presented as *n* (%) and compared using the chi-square test of independence.

Table S6. Comparison of RSV vs. non-RSV infected patients (*n* = 573).

	**NRS (440)**			**IMV (133)**		
	**RSV**	**non-RSV**	***p***[†]	**RSV**	**non-RSV**	***p***[†]
N (%)	261 (59)	179 (41)	-	88 (66)	45 (34)	-
Age at PICU admission						
Median (IQR), mo	4 (1.9-8)	8 (4-13)	**<0.0001**	2 (1.3-5.8)	4 (2-10)	**0.007**
Weight (kg), median (IQR)	6.3 (4.9-8.4)	8.1 (5.7-10)	**<0.001**	5.3 (3.8-7.2)	5.2 (4.4-8.4)	0.42
Female sex, *n* (%)	109 (42)	78 (43)	0.71	42 (48)	20 (44)	0.72
Gestational age at delivery, median (IQR), week	38 (36-38)	38 (36-38)	0.72	37 (35-38)	36 (31-38)	0.42
PICU LOS (d), median (IQR)	3 (2-4)	2 (1-3)	**<0.0001**	8 (6-10)	8 (4.5-11)	0.85
PIM-III ROM, % (IQR)	1.0 (0.9-1.2)	1.0 (0.9-1.3)	0.69	1.0 (0.9-1.4)	1.2 (1-3.4)	**0.03**
Intubation duration (d), median (IQR)	n/a	n/a		7 (5-9)	6 (4.5-11)	0.93
Ventilator-free-days, median (IQR)	n/a	n/a		21 (19-23)	22 (18-23)	0.94
Antibiotic						
Usage, n (%)	75 (29)	51 (28)	0.96	83 (94)	42 (93)	0.82
Duration (d), median (IQR)	7 (4-10)	7 (2-10)	0.71	7 (7-10)	7 (6.5-10)	0.27

NRS = noninvasive respiratory support, IMV = invasive mechanical ventilation, PICU = pediatric intesnvie care unit, IQR = interquartile range, mo = months, PICU LOS = pediatric intensive care unit length of stay, PIM-III ROM = Pediatric Index of Mortality-III Risk of Mortality, d = days, n/a = not applicable; [†] Continuous variables

are presented as median (interquartile range) and compared using the Wilcoxon rank-sum test; categorical variables are presented as *n* (%) and compared using the chi-square test of independence. Bold highlights *p* values < 0.05.

Table S7. Antibiotic Usage, Lab Results for Early Intubation Group only (*n* = 96).

	**Prior to admission (*n* = 52)**	**After admission (*n* = 44)**	***p*†**
Total, *n* (%)	49 (94)	41 (93)	0.83
Duration (d), median (IQR)	7 (4.5-9.5)	10 (7-10)	**0.007**
Time to initiation (h after intubation), median (IQR)	0 (0-4.75)	7 (7-34)	**<0.001**
WBC (x 1,000), median (IQR)	9.4 (6.8-12)	8.6 (6.3-12)	0.44
% band, median (IQR)	2 (0-13)	0.5 (0-7.75)	0.52
Procalcitonin, median (IQR)	2.6 (0.3-18)	0.9 (0.3-2.9)	0.22

d = days, IQR = interquartile range, h = hours, WBC = white blood cell; † Continuous variables are presented as median (interquartile range) and compared using the Wilcoxon rank-sum test; categorical variables are presented as *n* (%) and compared using the chi-square test of independence. Bold highlights *p* values < 0.05.

A

	OR	Lower 95%	Upper 95%	*p*
Weight	0.82	0.75	0.89	**<0.001**
Gestational Age: <=36	1.89	1.23	2.86	**0.003**
Pre-existing Neurologic or Genetic Conditions	4.77	2.54	8.95	**<0.001**
Pathogen: Single	1.96	0.43	8.90	0.384
Pathogen: Double	13.04	2.86	59.57	**0.001**
Pathogen: ≥ 3	102.48	20.46	513.41	**<0.001**

B

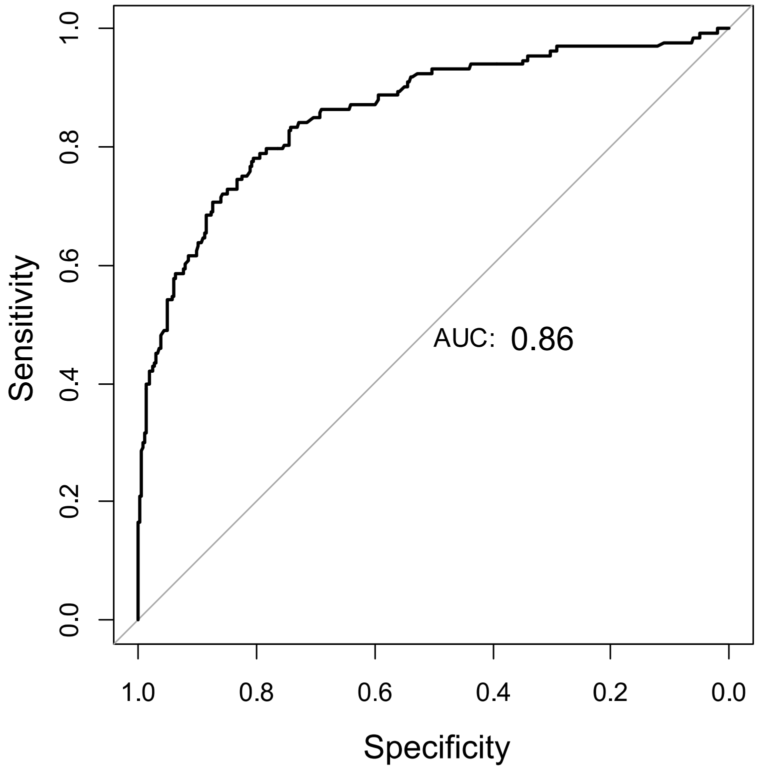

Figure S2. Predictive model for critical bronchiolitis patients requiring invasive mechanical ventilation using pathogen data. (A) Table of variables inclusive of pathogen load used to generate the predictive model. (B) Receiver Operating Characteristic (ROC) curve generated from chosen variables. AUC = area-under-the curve
